# Prehospital administration of blood products: experiences from a Finnish physician-staffed helicopter emergency medical service

**DOI:** 10.1186/s12873-020-00350-x

**Published:** 2020-07-07

**Authors:** Pauli Vuorinen, Joonas-Eemeli Kiili, Piritta Setälä, Antti Kämäräinen, Sanna Hoppu

**Affiliations:** 1 Emergency Medical Services, Tampere University Hospital, PO Box 2000, FI-33521 Tampere, Finland; 2 Faculty of Medicine and Health Technology, Tampere University, PO Box 2000, FI-33520 Tampere, Finland; 3 Department of Emergency Medicine, Hyvinkää District Hospital, Hyvinkää, Finland

**Keywords:** Prehospital blood products, Red blood cells, Freeze dried plasma, Helicopter emergency medical services, HEMS

## Abstract

**Background:**

Massive infusions of crystalloids into bleeding hypotensive patients can worsen the outcome. Military experience suggests avoiding crystalloids using early damage control resuscitation with blood components in out of hospital setting. Civilian emergency medical services have since followed this idea. We describe our red blood cell protocol in helicopter emergency medical services (HEMS) and initial experience with prehospital blood products from the first 3 years after implementation.

**Methods:**

We performed an observational study of patients attended by the HEMS unit between 2015 and 2018 to whom packed red blood cells, freeze-dried plasma, or both were transfused. The Student’s two-sided T-test was used to compare vitals in prehospital phase with those at the hospital’s emergency department. A *p*-value < 0.05 was considered significant.

**Results:**

Altogether, 62 patients received prehospital transfusions. Of those, 48 (77%) were trauma patients and most (*n* = 39, 81%) suffered blunt trauma. The transfusion began at a median of 33 (IQR 21–47) minutes before hospital arrival. Median systolic blood pressure showed an increase from 90 mmHg (IQR 75–111 mmHg) to 107 mmHg (IQR 80–124 mmHg; *P* < 0.026) during the prehospital phase. Four units of red blood cells were handled incorrectly when unused red blood cells were returned and required disposal during a three-year period. There were no reported adverse effects from prehospital transfusions.

**Conclusion:**

We treated two patients per month with prehospital blood products. A prehospital physician-staffed HEMS unit carrying blood products is a feasible and safe method to start transfusion roughly 30 min before arrival to the hospital.

**Trial registration:**

The study was retrospectively registered by the Tampere University Hospital’s Medical Director (R19603) 5.11.2019.

## Background

Prompt transfusion of whole blood to control bleeding was highlighted more than a century ago when warm, fresh whole blood was drawn and transfused early after trauma [1]. During the 1970s, fluid resuscitation was begun using normal saline solution and gradually moved towards blood component therapy [2]. This followed the idea that organ perfusion is restored when hypovolemia is reversed.

Delayed crystalloid infusion in the prehospital phase is beneficial for patients with penetrating injuries [3]. The association between acute traumatic coagulopathy at arrival in the trauma centre and the volume of prehospital crystalloid infusion is well established [4]. Studies published during the last 10 years have made it evident that haemostatic resuscitation of bleeding trauma patients with early deployment of blood components is now state of the art [5–9].

London’s Air Ambulance was one of the first civilian emergency medical services (EMS) to introduce prehospital blood transfusions in 2012 [10]. Since then, several emergency medical service agencies mainly in metropolitan cities have adopted prehospital packed red blood cells (PRBCs) as well as prehospital plasma. The majority of reports justify the use of prehospital blood products with decreased time to transfusion, but only scene times and transport times are published [11–14]. The prehospital physician-staffed helicopter emergency medical services (HEMS) unit in Tampere University Hospital Specific Catchment Area serves smaller number of inhabitants populated far more sparsely than most emergency medical services publishing prehospital blood product usage. We describe here our initial experiences with prehospital blood product implementation since we believe there are several emergency medicine service agencies still considering need of prehospital blood product implementation. A special emphasis was put on the exact time elapsed from the transfusion commencement to hospital arrival.

## Methods

### Design

We collected data from all patients transfused with prehospital blood products from November 2015 until October 2018. Their medical records were reviewed for units of blood products transfused, start time of prehospital transfusion, first vital signs measured by the paramedics, first vital signs at the emergency department, ward of admission after the emergency department, and whether the patient was discharged alive from the university hospital. According the Finnish law, the patient consent and the statement from the Ethics Committee were waived as this observational study was based on medical records and no interventions were conducted. The study was approved by the Tampere University Hospital’s Medical Director.

### Setting

The HEMS crew operating 24/7 consists of a prehospital physician with a specialist degree in anaesthesiology and intensive care medicine, a HEMS-nurse-paramedic, and a helicopter pilot. They are dispatched by the national dispatch service centre to all major trauma in the area of Tampere University Hospital and parts of neighbouring regions totalling 1,000,000 inhabitants. The HEMS unit is also dispatched on EMS missions concerning out-of-hospital cardiac arrests, imminent childbirth, acute poisoning, and, at the discretion of the prehospital physician on call, any other life-threatening emergency in the area. The yearly number of HEMS missions is around 3000, of which one-third are due to trauma.

Two units of type O- packed red blood cells produced from voluntary blood donations by the Finnish Red Cross Blood Service were taken on board from November 2015 onwards, and two units of freeze-dried plasma (FDP, Lyoplas AB, Deutsches Rotes Kreutz, Blutspendedienst West, Hagen, Germany) were added in April 2017. Standard operating procedure recommends the prehospital physician use PRBCs and freeze-dried plasma in cases of major trauma with suspected significant haemorrhage, e.g., systolic blood pressure under 90 mmHg or absent radial pulse. Major bleeding without preceding trauma—e.g., postpartum haemorrhage or a ruptured aortic aneurysm—was also considered a viable case for prehospital blood product transfusion. The standard operating procedure also recommends giving the patient 1 g of tranexamic acid intravenously (20 mg/kg for children) when making the decision to use prehospital blood products. After commencing the infusion, the prehospital physician fills out a prespecified form that has an identifier tag from the PRBC and from the freeze-dried plasma bottle with the time of transfusion and a comment field for if any immediate transfusion complications occurred. The HEMS crew receives new PRBCs when they present a copy of the completed form at the hospital’s blood service.

The type O- packed red blood cells are stored in a Credo Promed (Pelican BioThermal, Plymouth, USA) temperature-controlled insulated box that thermally protects the PRBC for up to 72 h. To ensure the quality of the PRBCs, the cold elements are changed every 48 h. PRBCs are replaced with fresh units weekly irrespective of their usage. The university hospital’s blood service accepts unused PRBCs for further use at no expense. The HEMS is charged 128.23 euros per used PRBC unit. Every unit encloses a data logger (TempTale4, Sensitech Inc., Beverly, USA) to track the surrounding temperature. The temperature in the city of Tampere during the data collection period ranged from − 26.8 °C (January 7th, 2016) to + 31.4 °C (July 17th, 2018). The PRBCs are infused through battery-powered fluid warmer (Buddy Lite™, Belmont Medical Technologies, Billerica, USA).

### Statistics

Microsoft Excel 2016 spreadsheet and statistical program (Microsoft Corporation, Redmond, USA) was used to analyse the data. Due to skewness of the data continuous variables are presented as median and interquartile range (IQR). Exceptionally, the average amount of prehospital blood product transfusions started is represented as a mean and standard deviation (SD) as it is more descriptive. A two-sided paired T-test was used to compare patients’ vital signs and relative risk (RR) with a confidence interval (CI) that was used for categorical comparisons. A probability of less than 0.05 was considered significant.

## Results

**Fig. 1:**
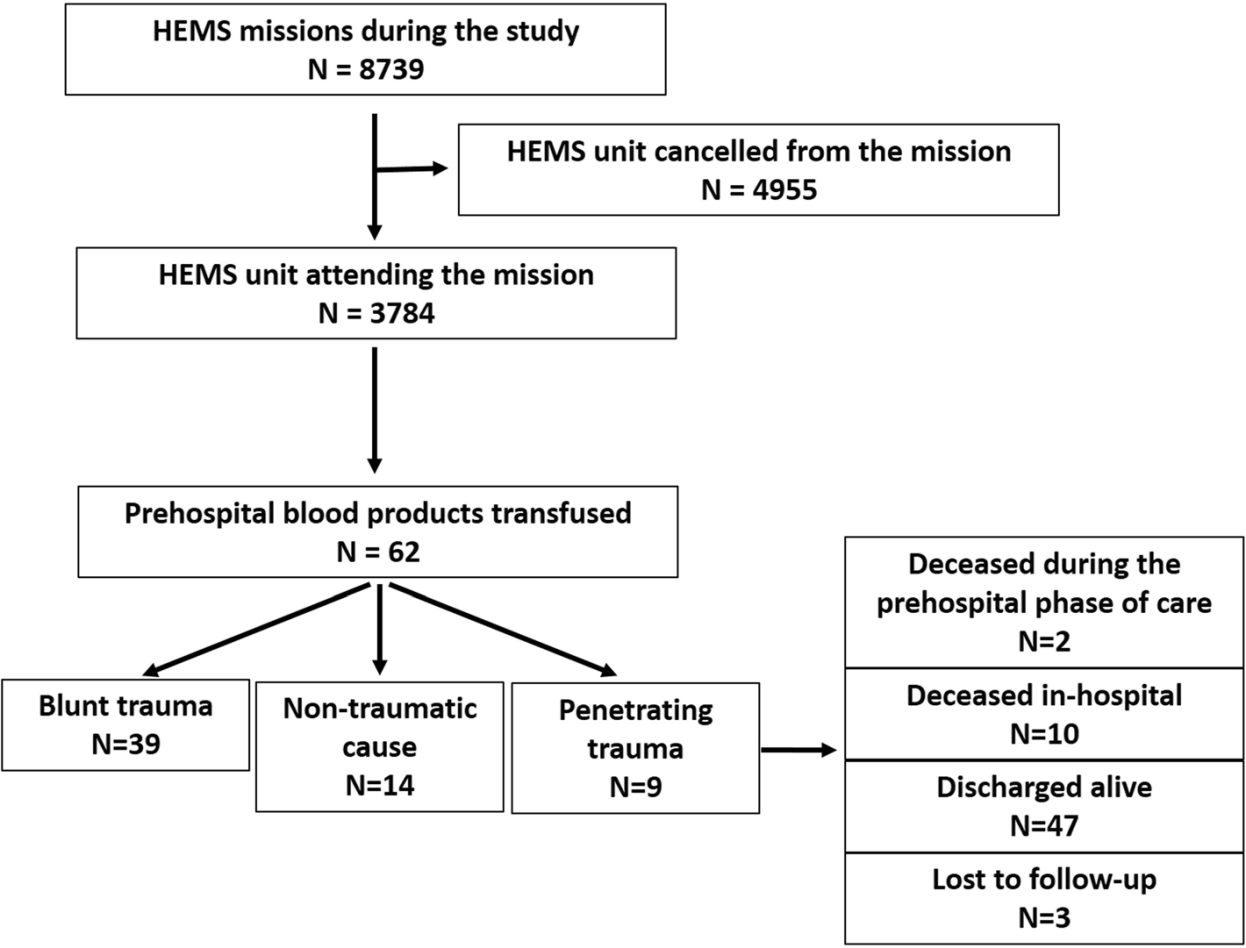
Flow chart of patients. HEMS: Helicopter emergency medical services

Out of 8739 HEMS missions altogether 62 (0.7%) patients received prehospital blood products. Forty-six (74%) of them were male, and the median age was 47 (IQR 28–67) years. Fig. 1 shows the patient flow. The leading cause (*n* = 32, 52%) of HEMS missions was road traffic accidents, followed by other trauma—i.e. violence (*n* = 8), an accidental gunshot wound (*n* = 1), and falls from heights (*n* = 3). In total, 39 (81%) of trauma patients suffered blunt trauma. Non-traumatic causes for prehospital blood transfusion were gastrointestinal bleeding (*n* = 6), aortic disease (*n* = 4), postoperative complications (*n* = 3), and one patient who presented with placental ablation.

Thirty-four patients were given one unit of PRBC and 27 patients received two units. On one mission, the HEMS crew was equipped with four PRBC units and every bag was transfused to one victim of severe stab injuries. The mean amount of PRBC per patient was 1.5 (SD 0.6) units. Following the implementation of freeze-dried plasma, 38 patients were given PRBC and 18 (47%) of them were transfused with 1.5 (SD 0.5) units of plasma as well. The decision to use freeze-dried plasma was made more often when the patient was given two units of PRBC than when transfusing only one unit (RR 3.64; 95% CI 1.26–10.49). The median systolic blood pressure increased from 90 mmHg (IQR 75–111) at the beginning of transfusion to 107 mmHg (IQR 81–124) at the emergency department (*P* < 0.026). Altogether, 55 patients (89%) were injected with tranexamic acid along with prehospital blood products.

The median delay from the emergency call to the beginning of prehospital transfusion was 75 min (IQR 53–97). It took a median of 19 min (IQR 10–31) to commence the transfusion after meeting the patient. The transfusion began 33 min (IQR 21–47) before arrival at the emergency department. For three patients, the transfusion of prehospital PRBC began at the emergency department. Two patients succumbed to their injuries in the prehospital phase and ten patients who survived to the receiving hospital were never discharged (Fig. 1). This accounts for a short-term mortality of 21%. Only six patients arrived at the hospital within an hour of the emergency call. Patient characteristics are shown in Table 1.

**Table 1:** Patient characteristics

Male		46 (74%)
Median age (IQR) (years)		47 (28–67)
Cause of transfusion	Blunt trauma	39 (63%)
	Penetrating trauma	9 (14%)
	Non-trauma	14 (23%)
Patients transfused 1 PRBC		34
	1 FDP	3
	2 FDP	1
Patients transfused 2 PRBC		27
	1 FDP	6
	2 FDP	8
Median pretransfusion systolic blood pressure (IQR) (mmHg)		90 (75–111)
Median pretransfusion heart rate (IQR) (1/min)		93 (80–110)
Median emergency room systolic blood pressure (IQR) (mmHg)		107 (81–124)
Median emergency room heart rate (IQR) (1/min)		100 (90–115)
Median delay in minutes from emergency call to start of transfusion (IQR)		71 (53–87)
Median delay in minutes to reach hospital after start of transfusion (IQR)		33 (21–47)
Patient care after emergency department (*n* = 56)	ICU	12 (21%)
	Operating room	36 (64%)
	Normal hospital ward	8 (14%)

*IQR* interquartile range; *PRBC* packed red blood cells, *FDP* freeze-dried plasma; *mmHg* millimetres in mercury; *ICU* intensive care unit

Of the 57 patients admitted to the Tampere University Hospital, blood product transfusion was continued for 37 patients (65%) and of those patients massive transfusion protocol was activated for 20 patients (35%). Twelve patients (21%) were admitted to the intensive care unit (ICU) after the emergency department and 36 patients (64%) required emergency surgery or radiological interventions. Forty-seven (82%) of the patients surviving to the Tampere University Hospital were discharged alive either home or to another health care facility. For patients surviving to discharge, the median length of stay in the ICU was 3 days (IQR 1–6) and the median length of stay in hospital was 8 days (IQR 5–16). There were no transfusion-related complications concerning the prehospital blood products. The data loggers showed the red blood cells remaining constant in temperature in the insulated box regardless of the surrounding temperature during operative use. An unfortunate communication failure caused wastage of four unused PRBC units. This means that less than 1% of the red blood cell units handled were wasted.

## Discussion

We were the first HEMS unit in Finland to deploy a protocol for continuously carrying prehospital red blood cells on EMS missions. Prehospital red blood cells were used on 0.7% of HEMS missions, which equals to two patients per month. The transfusion began 33 min before arrival to the hospital.

Previous descriptions from civilian emergency medical services utilizing prehospital blood products come from Great Britain [10, 11], Australia [12, 15], Norway [16], the Netherlands [17], and Sweden [18]. The collective conclusion from these reports, including ours, is that it is possible to deliver prehospital transfusions in a safe manner. Depending on the surrounding population 1–8 patients per month are in need of prehospital transfusion by a HEMS unit [10, 11, 15–18]. Rehn et al. stated that 5% of patients in their trauma databank presented with suspected major haemorrhage [14]. Zielinski et al. estimated that 1–2% of Norwegian HEMS units’ patients are in need of prehospital transfusions [16] and Sato-Folatre present a figure of 2.5% from Sweden [18]. We used prehospital transfusion on 0.7% of our HEMS missions, which is somewhat less than other reporting HEMS units; however, this was still consistent since there were differences in mission profiles.

None of the previously mentioned articles reported immediate transfusion complications and neither did we. Dalton reported of one patient out of 112 with transient breathing difficulties after infusing 5 l of crystalloid with PRBCs [19]. Barkana et al. described one soldier out of 40 with a fine truncal rash in the hospital phase after a prehospital transfusion [20]. Thus, it is reasonable to say that prehospital transfusions introduce no excessive threat to patient care.

Our patients presented the same characteristics as earlier reports from civilian emergency medicine services: predominantly adult males suffering blunt trauma from road traffic accidents [11–14]. The 24-h mortality of our patients is within the range summoned and published by Rijnhout et al. [21]. Regarding the prehospital time frame, Sperry et al. stated that prehospital transfusions would not delay the patient reaching the hospital [13]. Rehn et al., on the other hand, found a delay of 7 min in patients receiving prehospital transfusions and arriving alive at major trauma centres compared to patients not transfused during the prehospital phase of care [14]. Even if the use of prehospital blood products would delay the patient some minutes from reaching the hospital, we consider the possibility to spare ca. half an hour in the process of starting a transfusion highly valuable. There are no type O- PRBCs stored at the emergency department of Tampere University Hospital. Emergency transfusion of type O- PRBCs or massive transfusion protocol needs to be activated with dedicated software and further confirmed by phone. The onset of transfusion can take some minutes after the phone call. This is why three patients received their first blood products from the HEMS crew even after arrival at the hospital. Powell et al. stated that as little as a 10-min postponement in the transfusion decreases the odds of survival for a trauma patient [22]. Since our study has no control group, we are unable to show if prehospital transfusion had any effect on mortality or morbidity. In an extensive systematic review, there was a significant reduction in the odds of long-term mortality when PRBCs and plasma were use but data quality was considered low and a craving for controlled trials exists [21]. The percentage of patients receiving further transfusions in the emergency department was consistent between our findings and those reported by Lyon et al. [11].

The CRASH-2 trial showed a clear advantage of promptly administered tranexamic acid to bleeding trauma patients [23]. We recognise the benefit to the patient, ease of use and relative safety of antifibrinolytic agents and yet more than 10% of patient never received their dose in the prehospital phase of care. There are many things happening at the same time in cramped circumstances while transferring a bleeding patient to the hospital. The workload of personnel in the emergency medical services is evidently higher when a new treatment is implemented and forgetfulness is humane. We noticed this with the very first patients receiving PRBCs without tranexamic acid and added an extra checklist in the insulated box with the blood cells. Unfortunately, tranexamic acid was dismissed after this as well. In comparison, mean time of tranexamic acid administration in CRASH-2 trial was slightly under 3 h [23] and in further study, CRASH-3, in patients with traumatic brain injury 72% were assigned within 3 h of injury [24].

Weaver et al. presented an abstract at the London Trauma conference in 2012 in which they described a red blood cell breach in the return phase of logistics. Ironically, this was exactly the case in our unit when the personnel at the hospital blood service failed to take care of the unused red blood cells in time and the temperature rose too high. Heschl et al. report a wastage of 0.5% [12], Bodnar et al. 1.6% [15], and Holcomb et al. 1.9% [25]. The percentage is quite low, but every effort should be made to minimize the wastage.

We present a rather small number of patients in this article, but due to the PRBC protocol, we are sure every patient transfused with prehospital blood products in our area was included. On the other hand, we have no way to verify if we encountered every patient in need of prehospital transfusion. The small number of patients prevents extensive comparisons between different patient groups. Since there was no need to visit the hospital’s blood service on our way to the EMS mission, the delay to the start of transfusion was minimized, and because of the prespecified form we were able to recollect the time of transfusion.

## Conclusion

We encountered two patients per month in need of prehospital transfusions and were able to overcome the logistical challenges of keeping the red blood cells at the correct temperature. A physician-staffed HEMS unit is a feasible and safe way of decreasing the time to transfusion in critically injured or ill patients.

## Data Availability

The datasets used and/or analysed during the current study are available without personifying information from the corresponding author on reasonable request.

## Abbreviations

CI: Confidence interval
EMS: Emergency medical services
FDP: Freeze-dried plasma
HEMS: Helicopter emergency medical services
ICU: Intensive care unit
IQR: Interquartile range
PRBCs: Packed red blood cells
RR: Relative risk
SD: Standard deviation
